# Psychiatric Safety Signals of GLP-1 Receptor Agonists: A FAERS-Based Pharmacovigilance Study with Explainable Machine Learning

**DOI:** 10.3390/ph19060953

**Published:** 2026-06-18

**Authors:** Suhyeon Moon, EunJu Lee, Doyeon Kim, Kyung Hee Choi, Yeo Jin Choi, Sooyoung Shin

**Affiliations:** 1 Department of Biohealth Regulatory Science, Graduate School, Ajou University, Suwon 16499, Republic of Korea; 2 Department of Pharmacy, College of Pharmacy, Gachon University, Incheon 21936, Republic of Korea; 3 College of Pharmacy and Institute of Integrated Pharmaceutical Science, Kyung Hee University, Seoul 02447, Republic of Korea; 4 Department of Pharmacy, College of Pharmacy, Ajou University, Suwon 16499, Republic of Korea

**Keywords:** GLP-1 receptor agonists, pharmacovigilance, FAERS, psychiatric adverse events, disproportionality analysis, machine learning, SHAP

## Abstract

**Background:** As glucagon-like peptide-1 (GLP-1) receptor agonist use expands globally, reports of psychiatric adverse events (AEs) have increased in spontaneous reporting databases. However, which case-level characteristics are associated with these reporting patterns remains insufficiently characterized. This study aimed to characterize case-level features associated with psychiatric AE reporting in GLP-1 receptor agonist users through pharmacovigilance and explainable machine learning methods. **Methods:** The FDA Adverse Event Reporting System (FAERS) data (2021 Q2–2025 Q3) were analyzed using a comparator-based approach comprising other antidiabetic and anti-obesity agents. Disproportionality analyses (PRR, ROR, and IC) were performed to detect consolidated safety signals at the Preferred Term (PT) level, with additional drug-specific analyses for individual GLP-1 receptor agonists. Three classification models (logistic regression, XGBoost, and LightGBM) were developed, and SHAP values were used to identify case-level features associated with psychiatric AE reporting patterns. **Results:** A total of 211,195 unique cases were included (111,105 for GLP-1 receptor agonists; 100,090 for comparators). Sixteen PTs met consolidated signal criteria, with suicidal ideation being the most frequently reported (ROR 2.95). Drug-specific analyses indicated that signal magnitudes were consistently higher for semaglutide than tirzepatide. The XGBoost model achieved an AUROC of 0.816. SHAP analysis showed that age ≥65 years had the highest mean |SHAP| value (0.57) with a negative direction, corresponding to a lower predicted probability of psychiatric AE reporting in older adults. Semaglutide use ranked second (0.35) and showed a positive direction. Absence of concomitant medications (0.20) and diabetes indication (0.10) showed negative directions, while younger age (19–44 years, 0.08) showed a positive direction. These patterns remained consistent in sensitivity analysis excluding concomitant psychotropic medication users (AUROC 0.797). **Conclusions:** Older age status was associated with decreased predicted probability of psychiatric AE reporting, while semaglutide use and younger age showed positive contributions. These case-level patterns, identified through pharmacovigilance analysis using a comparator-based approach and explainable machine learning, suggest that reporting patterns may differ across subgroups and that the observed reporting heterogeneity among younger adults and semaglutide users merits further investigation in prospective studies.

## 1. Introduction

Obesity and type 2 diabetes mellitus represent major public health burdens worldwide, contributing substantially to chronic metabolic and cardiovascular morbidity and mortality [1,2,3]. The global prevalence of both conditions has continued to increase across all regions and age groups, with obesity rates rising markedly among adults and younger populations, and type 2 diabetes projected to affect over 1.3 billion individuals by 2050 [3,4]. The increasing prevalence of both obesity and type 2 diabetes has heightened the need for pharmacotherapies that address both glycemic control and weight management. Glucagon-like peptide-1 (GLP-1) receptor agonists have emerged as a major therapeutic class spanning both indications, demonstrating substantial efficacy in weight reduction and durable improvements in metabolic control across clinical trials and real-world practice [5,6,7]. Originally developed for glycemic control in type 2 diabetes, GLP-1-based therapies have subsequently expanded into chronic weight management, with liraglutide, semaglutide, and more recently tirzepatide demonstrating substantial therapeutic benefits in both metabolic settings [8,9]. The scope of their approved indications continues to broaden; semaglutide recently became the first anti-obesity agent approved for cardiovascular risk reduction in adults with overweight or obesity [10]. As the clinical use of these agents grows across an increasingly diverse patient population, a comprehensive understanding of their safety profile relative to other established treatments for diabetes and obesity remains critical.

GLP-1 receptors are distributed not only in peripheral metabolic tissues but also throughout the central nervous system [11,12]. These receptors are expressed in hypothalamic, brainstem, and limbic regions, including the amygdala, hippocampus, and nucleus accumbens, suggesting a role in the regulation of appetite, emotion, and reward processing [13,14,15,16]. These biological interactions have raised concerns regarding the potential for GLP-1 receptor agonists to be associated with psychiatric adverse events (AEs), distinct from the psychopathology of underlying metabolic conditions [17]. The extent of blood–brain barrier penetration and central receptor engagement may also differ across individual GLP-1 receptor agonists [15,18]. This raises the possibility that psychiatric safety may not be uniform across the drug class.

Current evidence regarding the psychiatric safety of GLP-1 receptor agonists remains inconclusive. Major randomized controlled trials, including the SUSTAIN and STEP programs, did not identify a consistent increase in psychiatric AEs [12,19,20], although these trials were generally not powered for psychiatric endpoints and often excluded patients with pre-existing psychiatric conditions. Findings from post-marketing surveillance and real-world observational studies have been heterogeneous. A large community-based cohort study of over 160,000 matched patients with obesity reported that GLP-1 receptor agonist users had higher rates of major depression, anxiety, and suicidal behavior compared with non-users [21]. In contrast, an analysis of electronic health records involving over 240,000 patients found that semaglutide was associated with a lower risk of both incident and recurrent suicidal ideation compared with other anti-obesity medications [22]. A recent new-user, active-comparator cohort study also reported a modest but statistically significant increase in incident depression among GLP-1 receptor agonist initiators compared with sodium-glucose cotransporter-2 (SGLT-2) inhibitor initiators [23]. Meanwhile, the U.S. Food and Drug Administration (FDA) concluded in January 2026 that current evidence does not demonstrate a causal link between GLP-1 receptor agonists and suicidal ideation, and requested the removal of suicidal ideation and behavior warnings from the labeling of liraglutide, semaglutide, and tirzepatide, following a comprehensive meta-analysis of 91 clinical trials involving over 107,000 patients and a large retrospective cohort study using the Sentinel System [24].

Several pharmacovigilance studies have examined the psychiatric safety of GLP-1 receptor agonists using the FDA Adverse Event Reporting System (FAERS) or other spontaneous reporting databases, identifying signals including nervousness, eating disorders, depression, anxiety, and suicidal ideation, with some studies suggesting drug-specific differences, particularly more prominent signals for semaglutide than for other agents in the class [25,26,27,28]. Most of these analyses used the entire FAERS database as the background reference and focused on aggregate-level signal detection [29,30].

This study aimed to characterize psychiatric AE reporting patterns associated with GLP-1 receptor agonists in comparison with other antidiabetic and anti-obesity agents using data from FAERS. We employed a comparator-based approach within the disproportionality analysis framework to better account for confounding by indication, conducted drug-specific disproportionality analyses to compare signal magnitudes across individual agents, and integrated explainable machine learning methods using SHapley Additive exPlanations (SHAP) to quantify the contribution of individual features at the case level and identify case-level characteristics associated with psychiatric AE reporting [31,32].

## 2. Results

### 2.1. Study Population

**Figure 1:**
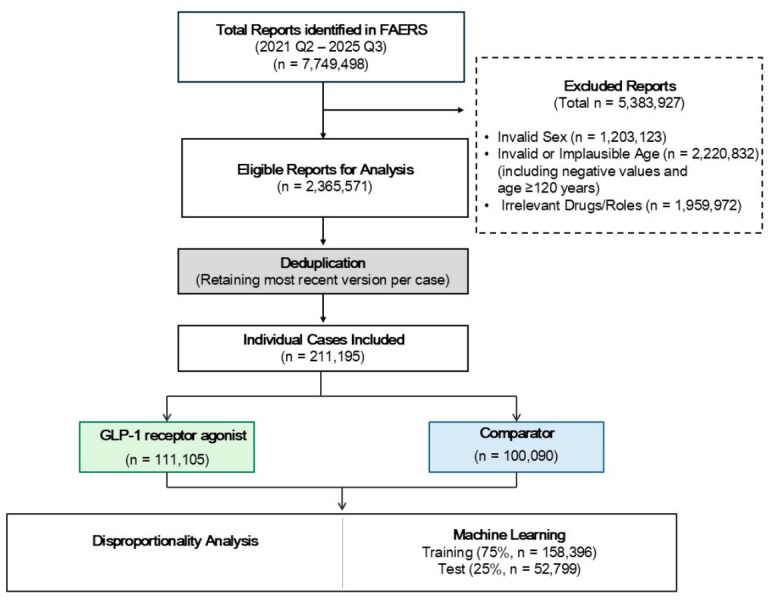
Flowchart of study population selection and data processing. Green and blue boxes denote the GLP-1 receptor agonist and comparator groups, respectively. Abbreviations: FAERS, FDA Adverse Event Reporting System; GLP-1 RAs, glucagon-like peptide-1 receptor agonists.

Between the second quarter of 2021 and the third quarter of 2025, a total of 7,749,498 reports were identified in the FAERS database. After excluding 5,383,927 reports due to invalid sex, invalid or implausible age (including negative values and age ≥120 years), or irrelevant drug roles, 2,365,571 reports were initially screened for further processing. Subsequent deduplication, which involved retaining only the most recent version of each case, yielded 211,195 unique cases. These included 111,105 cases in the GLP-1 receptor agonist group and 100,090 in the comparator group. This dataset was then divided into a training set of 158,396 cases (75%) and a test set of 52,799 cases (25%) for model development and evaluation (Figure 1).

### 2.2. Baseline Characteristics

Baseline characteristics are presented in Table 1. Female patients accounted for 70.3% of the GLP-1 receptor agonist group, compared with 53.7% in the comparator group. The GLP-1 receptor agonist group was younger overall, with middle-aged individuals (45–64 years) comprising 47.2% of cases, while the comparator group was predominantly older adults (≥65 years: 55.0%). Consumer-submitted reports represented the largest proportion in both groups, accounting for 84.9% of GLP-1 receptor agonist cases and 49.2% of comparator cases. Serious AEs were more common in the comparator group (39.5% vs. 13.9%), as were mortality outcomes (7.7% vs. 1.1%). All between-group differences reached statistical significance (*p* > 0.001).

### 2.3. Disproportionality Signals for Psychiatric Adverse Events

A total of 16 preferred terms (PTs) met the consolidated signal criteria (Table 2). Suicidal ideation was the most frequently reported (*n* = 986, ROR 2.95 [95% confidence interval (CI) 2.62–3.32]), followed by depression suicidal (ROR 2.32), self-injurious ideation (ROR 2.47), and suicidal behavior (ROR 4.24). Bipolar disorder showed a markedly elevated signal (ROR 6.88). Among depressive and affective symptoms, major depression (ROR 5.46), anhedonia (ROR 4.73), feelings of worthlessness (ROR 6.44), self-esteem decreased (ROR 7.50), and emotional distress (*n* = 417, ROR 2.37 [2.00–2.80]) also met signal criteria. Mood swings (ROR 2.41), intrusive thoughts (ROR 31.44), agoraphobia (ROR 8.93), emotional poverty (ROR 57.96), alcoholism (ROR 3.41), and anorexia nervosa (ROR 2.54) were additionally identified. No consolidated signals were detected for depressive disorders, anxiety disorders, or mood disorders at the class level.

**Figure 2:**
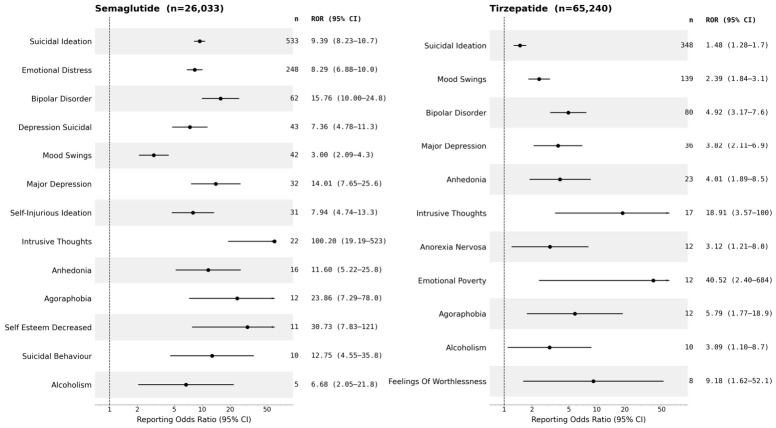
Forest plots of RORs for drug-specific psychiatric AEs associated with semaglutide and tirzepatide. Abbreviations: CI, confidence interval; ROR, reporting odds ratio; AEs, adverse events.

Drug-specific analyses indicated that suicidal ideation and bipolar disorder signals were consistently observed across semaglutide and tirzepatide, although the magnitude differed between agents (Figure 2). For semaglutide, suicidal ideation (*n* = 533, ROR 9.39 [8.23–10.7]), bipolar disorder (ROR 15.76 [10.00–24.8]), and emotional distress (*n* = 248, ROR 8.29 [6.88–10.0]) were among the more prominent signals. Corresponding estimates for tirzepatide were lower in magnitude, including suicidal ideation (*n* = 348, ROR 1.48 [1.28–1.7]) and bipolar disorder (ROR 4.92 [3.17–7.6]).

### 2.4. Predictive Performance and Model Selection

**Figure 3:**
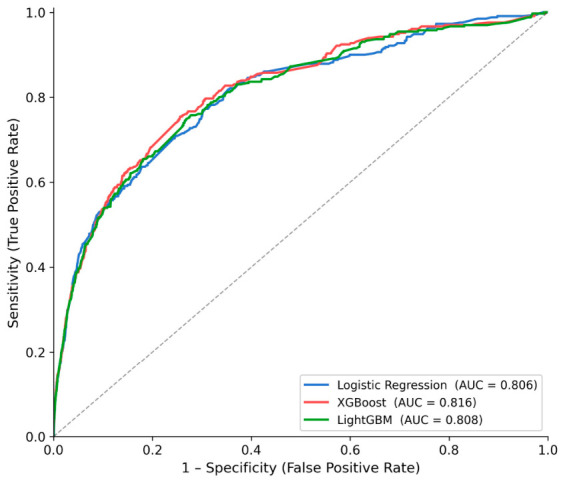
Comparison of ROC curves across prediction models. Abbreviations: ROC, receiver operating characteristic; AUC, area under the curve; XGBoost, extreme gradient boosting; LightGBM, light gradient boosting machine.

The Area Under the Receiver Operating Characteristic Curve (AUROC) on the test set was 0.806 for logistic regression, 0.816 for XGBoost, and 0.808 for LightGBM (Figure 3). XGBoost demonstrated the highest discriminative performance and was selected for SHAP analysis. Top-k enrichment analysis showed that the top 1% of cases ranked by predicted probability (*n* = 527) captured 14.8% of all psychiatric AE cases, with a positive predictive value of 9.3% and a likelihood ratio of 14.9; at the top 10% threshold, 53.6% of cases were identified (Table 3).

### 2.5. Feature Contributions Identified by SHAP

**Figure 4:**
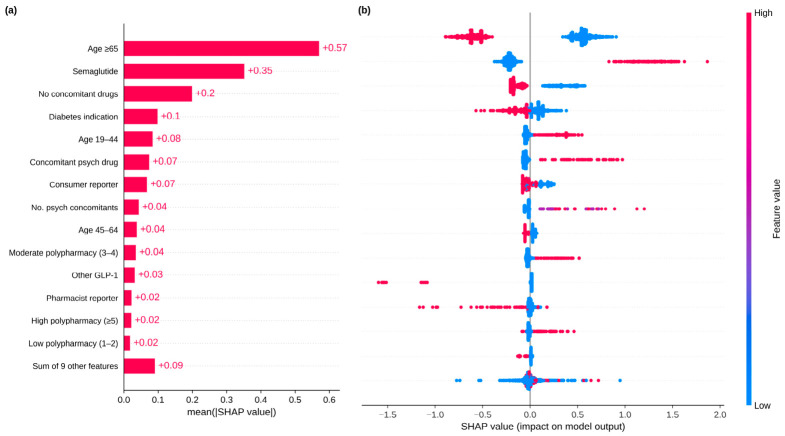
SHAP analysis of the XGBoost model. (**a**) Feature importance plot. (**b**) Beeswarm plot of feature effects on model predictions. Abbreviations: SHAP, Shapley Additive Explanations; XGBoost, extreme gradient boosting.

SHAP analysis of the XGBoost model showed that age ≥65 years had the highest mean |SHAP| value (0.57) with a negative direction, corresponding to a lower predicted probability of psychiatric AE reporting in older adults (Figure 4a,b). Semaglutide use ranked second (mean |SHAP| = 0.35) and showed a positive direction. Absence of concomitant medications (0.20) and diabetes indication (0.10) showed negative directions. Age 19–44 years (0.08), concomitant psychotropic drug use (0.07), and consumer reporter status (0.07) showed positive directions. Number of psychotropic concomitants (0.04), age 45–64 (0.04), and moderate polypharmacy (defined as 3–4 concomitant medications) (0.04) showed smaller contributions.

### 2.6. Results of Sensitivity Analyses

After excluding phentermine-containing products from the comparator group, the direction and magnitude of the primary disproportionality signals were largely preserved. Suicidal ideation retained a significant signal (ROR 3.12 [95% CI 2.77–3.52]), as did bipolar disorder (ROR 6.89 [4.57–10.39]). Fourteen of the 16 signals met all consolidated criteria; feelings of worthlessness and alcoholism did not reach significance due to reduced case counts (Supplementary Table S1).

When the disproportionality analysis was restricted to healthcare professional reports (GLP-1 RA: *n* = 121,545; comparator: *n* = 783,961), suicidal ideation (ROR 11.32 [9.51–13.48]), emotional distress (ROR 13.36 [9.94–17.94]), and depression suicidal (ROR 23.91 [11.21–51.00]) remained significant, with signal magnitudes generally higher than in the primary analysis (Supplementary Table S2).

Temporal trend analysis showed a notable increase in psychiatric AE reporting proportion for GLP-1 receptor agonists beginning in 2023 Q3, coinciding with the initiation of the European Medicines Agency (EMA) safety review (χ^2^ = 93.79, *p* < 0.001), while no comparable increase was observed in the comparator group (χ^2^ = 0.08, *p* = 0.78) (Supplementary Figure S1). Joinpoint regression further identified one inflection point at approximately 2023 Q4, with a significant increasing trend prior to the joinpoint (quarterly percent change [QPC] = +19.6%; 95% CI: 9.5–29.7%; *p* = 0.002), followed by a non-significant plateau thereafter (QPC = −2.8%; *p* = 0.74). No joinpoint was identified for comparators (QPC = −0.4%; *p* = 0.71) (Supplementary Table S3).

After excluding 7892 cases with concomitant psychotropic medication use (*n* = 203,303), the XGBoost model maintained an AUROC of 0.797. The directionality of key feature contributions remained consistent with the primary model; age ≥65 years retained the largest SHAP contribution with a negative direction, while semaglutide use, younger age (19–44 years), and tirzepatide use showed positive directions (Supplementary Figures S2 and S3).

Calibration curves for the XGBoost model before and after post hoc isotonic regression calibration are provided in Supplementary Figure S4.

## 3. Discussion

The present study employed a comparator-based approach within the disproportionality analysis framework and explainable machine learning to characterize psychiatric AE reporting patterns associated with GLP-1 receptor agonists. Using a comparator group of other antidiabetic and anti-obesity agents, we identified psychiatric safety signals across 16 PTs. These signals were largely preserved after excluding phentermine-containing products from the comparator group (14 of 16 signals retained) and were generally larger in magnitude when the analysis was restricted to healthcare professional reports. Drug-specific analyses indicated that signal magnitudes were consistently higher for semaglutide than tirzepatide across multiple psychiatric PTs. SHAP analysis of the XGBoost model (AUROC 0.816) showed that age ≥65 years and semaglutide use had the largest contributions to model discrimination. Older adult status, absence of concomitant medications, and diabetes indication were associated with lower predicted reporting probability, whereas semaglutide use, younger age (19–44 years), and consumer reporting were associated with higher predicted reporting probability. Together, these findings suggest that reporting patterns within the GLP-1 receptor agonist class vary across subgroups, and that combining comparator-based pharmacovigilance with explainable machine learning can provide a more granular characterization than aggregate signal detection alone.

The semaglutide-related psychiatric safety signals observed in this study were broadly consistent with previous pharmacovigilance reports [28,33]. By contrast, whereas earlier studies based on full-database background reporting rates generally reported weak or absent signals for tirzepatide [28,33], our analysis identified significant signals for both semaglutide and tirzepatide. One possible explanation is the difference in study design; because we used a comparator group consisting of other antidiabetic and anti-obesity agents, the relative reporting pattern for tirzepatide may differ from that observed in studies using the overall database as the reference. At the case level, SHAP analysis showed that semaglutide use had a larger SHAP contribution than tirzepatide, consistent with the drug-specific disproportionality results and supporting the internal consistency of the overall pattern. Although tirzepatide had a substantially larger overall report base than semaglutide (*n* = 65,240 vs. 26,033), psychiatric AE signal magnitudes were consistently lower for tirzepatide. However, because tirzepatide was approved later by the FDA (2022) [34] and has had less time to accumulate FAERS reports, differences in early user populations and reporting patterns may influence these findings and should be considered when interpreting between-drug differences. These findings are broadly consistent with a recent independent analysis of the WHO VigiBase database, which reported significantly increased RORs for suicidal ideation with semaglutide (ROR 5.82), liraglutide (4.03), and tirzepatide (2.25), supporting the reproducibility of the observed reporting patterns across independent pharmacovigilance systems [35].

The case-level patterns identified through SHAP analysis warrant further consideration. Older age status (≥65 years) showed the largest overall feature contribution, yet was associated with a decreased predicted probability of psychiatric AE reporting. One possible interpretation is that GLP-1 receptor agonists are more commonly prescribed to older patients for diabetes management, where the clinical and demographic context may differ from that of younger patients using these agents for weight reduction, potentially contributing to differences in psychiatric AE reporting patterns [36,37]. Of note, in spontaneous reporting data, the lower predicted reporting probability in older adults may also partly reflect reporting-level factors, such as differences in symptom coding or the prioritization of other concurrent AEs, rather than true differences in clinical risk alone. The negative contribution of diabetes indication in the SHAP analysis is consistent with this interpretation. In contrast, younger age (19–44 years), semaglutide use, and concomitant psychotropic drug use were each associated with increased predicted probability. Together, these findings suggest that reporting patterns within the GLP-1 receptor agonist class may vary across patient subgroups.

In the top-k enrichment analysis, cases ranked in the top 10% by predicted probability captured 53.6% of all psychiatric AE cases, with the top 1% alone accounting for 14.8% at a likelihood ratio of 14.9%. These findings suggest that predicted probability scores may help support prioritization in pharmacovigilance workflows by identifying reports more likely to contain psychiatric AEs. However, the overall event prevalence was low (0.63%), and the model was developed and evaluated entirely within FAERS, which may limit the generalizability of these findings to other reporting systems or clinical settings. The AUROC values should therefore be interpreted as reflecting the model’s discriminative ability within the FAERS dataset rather than as evidence of causal relationships.

It is important to delineate the distinct objectives of the two analytical approaches used in this study. The disproportionality analysis aimed to identify AEs reported at disproportionately higher frequencies for GLP-1 receptor agonists at the PT level. The machine learning analysis aimed to identify case-level characteristics of reports involving psychiatric AEs, with the primary focus on SHAP-based feature characterization rather than prediction performance. Our findings do not contradict the FDA’s January 2026 decision to remove suicidal ideation warnings [24], as disproportionality signals reflect reporting imbalances rather than causal risk.

The temporal trend analysis showed a notable increase in psychiatric AE reporting for GLP-1 receptor agonists following the initiation of the EMA safety review in 2023 Q3 [38], with no comparable increase observed in the comparator group, indicating that regulatory communications may have influenced reporting behavior. Joinpoint regression corroborated this pattern, identifying a single inflection point at approximately 2023 Q4, one quarter after the initiation of the EMA safety review (2023 Q3). A significant increasing trend was observed prior to the joinpoint and a non-significant plateau thereafter, while no inflection was detected for comparators, consistent with previous findings [39]. The concurrent market expansion of semaglutide and tirzepatide during the study period may also have contributed to the increasing reporting volume, independent of changes in the underlying risk profile. However, the sensitivity analysis restricted to healthcare professional reports confirmed that key signals, including suicidal ideation, remained significant and were larger in magnitude, suggesting that a genuine pharmacovigilance signal may coexist with a stimulated reporting component.

SHAP analysis showed that young adults (aged 19–44 years) and semaglutide users were subgroups with consistently higher predicted reporting probabilities, a pattern that persisted after excluding cases involving concomitant psychotropic medications. The FDA’s January 2026 decision may also influence future reporting patterns, and continued monitoring following this regulatory change remains necessary. The findings of the present study suggest that heterogeneity in reporting patterns across subgroups warrants further investigation in prospective pharmacoepidemiological studies.

### Limitations

Several limitations of this study should be considered. First, as with all analyses based on spontaneous reporting systems, causal inference cannot be drawn from the observed associations [40]; disproportionality signals and predicted reporting probabilities reflect reporting patterns within the database rather than clinical risk. Second, indication data in FAERS are incompletely recorded, and both disproportionality and SHAP-derived findings may have been influenced by differences in indication mix across patient subgroups that could not be fully accounted for in the present analysis. Third, drug-specific analyses for agents with limited numbers of reports yielded wide CIs and should therefore be interpreted cautiously. Fourth, residual confounding due to unmeasured psychiatric history cannot be excluded, despite adjustment for concomitant psychotropic medication use. Fifth, regulatory communications may have influenced psychiatric AE reporting patterns, as suggested by the temporal trend analysis. Sixth, the machine learning model was validated using a random holdout from the same FAERS dataset, without temporal or external validation. Seventh, this study relied exclusively on the FAERS database without cross-validation against other pharmacovigilance databases such as VigiBase or EudraVigilance, which limits generalizability and increases the potential for database-specific reporting bias; future multi-database studies are warranted. Finally, reporting biases inherent to spontaneous reporting systems, including underreporting and stimulated reporting, may have influenced the observed signal patterns, and the generalizability of the machine learning findings to other reporting systems or clinical settings remains uncertain [40,41].

## 4. Materials and Methods

### 4.1. Data Source

This retrospective pharmacovigilance study utilized quarterly data from FAERS, covering the period from the second quarter of 2021 to the third quarter of 2025. This timeframe was selected to coincide with the approval of Wegovy^®^ (semaglutide 2.4 mg) by the FDA in June 2021 [42]. Data processing was conducted in accordance with the FDA’s guidance on Good Pharmacovigilance Practices [43].

### 4.2. Exposure and Comparator Definition

Reports were classified according to the reported antidiabetic or weight-management drug. The GLP-1 receptor agonist group included cases involving semaglutide, liraglutide, dulaglutide, exenatide, lixisenatide, or tirzepatide. The comparator group consisted of cases involving other antidiabetic or anti-obesity agents to account for confounding by indication, as these patients share similar underlying metabolic profiles and clinical trajectories with GLP-1 receptor agonist users. This group included metformin, insulin, SGLT-2 inhibitors, dipeptidyl peptidase-4 (DPP-4) inhibitors, sulfonylureas, orlistat, and phentermine-containing products. Additionally, concomitant use of psychotropic medications, such as antidepressants, anxiolytics, antipsychotics, and mood stabilizers, was included to adjust for potential confounding by underlying neuropsychiatric conditions. This comparator definition has been used in prior FAERS-based pharmacovigilance studies [44,45]. Details are provided in Supplementary Table S4.

### 4.3. Case Inclusion and Variable Construction

Reports were limited to cases with valid demographic information (sex recorded as male or female and age >0). When multiple versions of the same case were available, only the most recent case version was retained [46]. Study drugs were identified from the DRUG file and restricted to reports in which the drugs were coded as primary or secondary suspects (PS/SS) [47]. Clinical variables were derived from the INDI, OUTC, and THER tables. Concomitant medications were identified from drugs coded as concomitant (role code “C”), and the number of concomitant drugs was calculated at the case level and categorized into four groups (0, 1–2, 3–4, and ≥5) [48]. To address potential confounding by underlying psychiatric conditions, concomitant use of psychotropic medications, including antipsychotic agents, was included as a covariate, as recent evidence highlights the potential influence of these factors on observed associations [22,49].

### 4.4. Disproportionality Analysis and Signal Detection

For the disproportionality analysis, the unit of analysis was the individual case. Analyses were performed at the MedDRA version 27.0 PT level. To avoid statistical bias, duplicate reports of the same PT for an individual patient were removed prior to the calculation of disproportionality indices [46]. Disproportionality analyses were conducted using the comparator group as the reference group. For each AE, we calculated three established indices: the proportional reporting ratio (PRR), the reporting odds ratio (ROR), and the information component (IC). These calculations were based on 2 × 2 contingency tables, with a 0.5 continuity correction applied to all cells to address potential zero-cell counts; the specific table structure and mathematical formulas are detailed in Supplementary Table S5 Signals were defined based on the following consolidated criteria: PRR ≥ 2 (χ^2^ ≥ 4, *n* ≥ 3); the lower bound of the 95% CI for ROR > 1; and the lower bound of the 95% CI for IC > 0. To ensure robustness, a consolidated signal was defined as an AE meeting the criteria for all three indices simultaneously [50,51]. To further characterize signal patterns at the drug level, disproportionality analyses were repeated for each individual GLP-1 receptor agonist (semaglutide, liraglutide, tirzepatide, and dulaglutide) using the same comparator group and signal criteria. As drug-specific strata may contain limited case numbers, these results should be interpreted with caution.

### 4.5. Machine Learning Models

To complement the disproportionality analysis and characterize case-level features associated with psychiatric AE reporting, three binary classification models were developed: logistic regression, extreme gradient boosting (XGBoost), and Light Gradient Boosting Machine (LightGBM) [52,53]. Logistic regression was included as an interpretable baseline, while the two gradient boosting models were selected for their established performance in high-dimensional, imbalanced pharmacovigilance datasets. A total of 23 input features were constructed spanning four domains: demographic characteristics (sex, age group), reporting context (reporter type), clinical profile (indication for obesity or diabetes, concomitant medication burden, concomitant psychotropic medication use), and individual GLP-1 receptor agonist type (semaglutide, liraglutide, tirzepatide, dulaglutide, or other agent). Each GLP-1 receptor agonist was encoded as a separate binary indicator to allow the model to capture drug-level differences in psychiatric AE reporting patterns, rather than treating the drug class as a single exposure variable. Detailed feature definitions are provided in Supplementary Table S6. The dataset was stratified into training (75%) and test (25%) sets using a random seed of 92. To address class imbalance, appropriate class re-weighting strategies were applied to each model: class_weight = ‘balanced’ was used for logistic regression [54], while positive scale weights were optimized for the gradient boosting models, XGBoost and LightGBM [55]. Hyperparameters were optimized via 5-fold cross-validated grid search on the training set. Full model specifications and hyperparameters are detailed in Supplementary Table S7.

### 4.6. Outcome Definition and Categorization

The target outcome for the machine learning analysis was defined as the presence of at least one of 16 psychiatric AEs identified as consolidated signals in the preceding disproportionality analysis (PRR ≥ 2, χ^2^ ≥ 4, *n* ≥ 3, ROR lower 95% CI > 1, IC lower 95% CI > 0), as detailed in Supplementary Table S8. Each case was classified as positive (label = 1) if any of these 16 PTs appeared in the reported AE list, and negative (label = 0) otherwise. This binary outcome was constructed at the case level, consistent with the unit of analysis used throughout the machine learning pipeline. The use of disproportionality-confirmed signals as the outcome definition integrates two distinct analytical objectives: (1) population-level signal detection via disproportionality analysis, and (2) case-level prediction via machine learning. This integration was designed to identify the specific case-level characteristics associated with the signals detected at the population level [56].

### 4.7. Performance Evaluation and Interpretability

Model performance was evaluated on the test set using the AUROC to assess the model’s ability to discriminate labels within the dataset. Additionally, precision, recall, and F1-scores were calculated based on the F1-max threshold, which maximizes the F1-score on the precision-recall curve, rather than a fixed 0.5 cutoff. To ensure model interpretability and identify the case-level features contributing to the model’s predictions, we employed an explainable machine learning approach using SHAP (SHapley Additive exPlanations) [57]. Beyond discrimination metrics, cases with the practical utility of the model for prioritizing cases with higher predicted reporting probability was assessed through a top-k enrichment analysis. Cases in the test set were ranked by predicted probability, and the concentration of positive cases within the top 1%, 5%, and 10% of ranked cases was quantified using recall, positive predictive value, and likelihood ratio positive. A calibration curve was constructed, and post hoc probability calibration using isotonic regression was additionally performed for comparison. The disproportionality, regression, and machine learning analyses were conducted in Python (version 3.12.12).

### 4.8. Sensitivity Analysis

To verify the robustness of our findings, the following sensitivity analyses were performed: (1) repeating the disproportionality analysis after excluding phentermine-containing products from the comparator group; (2) restricting the disproportionality analysis to healthcare professional reports; (3) a temporal trend analysis examining quarterly psychiatric AE reporting proportions for GLP-1 receptor agonists and the comparator group, with pre-communication (2021 Q2–2023 Q2) [38] and post-communication (2023 Q3–2025 Q3) [58] phases defined based on the EMA safety communication in July 2023, supplemented by joinpoint regression with Bayesian Information Criterion (BIC)-based model selection to estimate segment-specific quarterly percent changes with 95% CIs [59]; and (4) retraining the models on a subset excluding cases with concomitant psychotropic medication use. All model-based sensitivity analyses utilized the same hyperparameter optimization strategy as the primary analysis [60].

### 4.9. Ethics Approval

This study was approved as exempt by the institutional review board of Ajou University (IRB No. 202512-HB-EX-001).

## 5. Conclusions

This study identified psychiatric safety signals across 16 PTs in GLP-1 receptor agonist users compared with the comparator group, with suicidal ideation, bipolar disorder, and major depression among the more prominent findings. Drug-specific analyses indicated that signal magnitudes were higher for semaglutide than tirzepatide, though both agents met signal criteria when a comparator-based approach was applied. SHAP analysis of the XGBoost model identified age ≥65 years and semaglutide use as having the largest SHAP contributions to model discrimination for psychiatric AE reporting, with younger age and concomitant psychotropic drug use also showing positive associations. Temporal trends suggest an influence of regulatory communications on reporting, although signals remained significant in analyses restricted to healthcare professional reports. Despite the FDA’s January 2026 labeling change, subgroup heterogeneity warrants further evaluation and continued monitoring. This study highlights the methodological value of integrating comparator-based pharmacovigilance with explainable machine learning.

## Figures and Tables

**Table 1:** Baseline characteristics of FAERS reports by exposure group.

Variable	Level	GLP-1	Non-GLP-1	*p*-Value
Total no. of cases		111,105	100,090	-
Age group (years)	<19	491 (0.4%)	2860 (2.9%)	<0.001
	19–44	23,704 (21.3%)	9890 (9.9%)	
	45–64	52,453 (47.2%)	32,270 (32.2%)	
	≥65	34,457 (31.0%)	55,070 (55.0%)	
Sex	Female	78,056 (70.3%)	53,754 (53.7%)	<0.001
	Male	33,049 (29.7%)	46,336 (46.3%)	
Geographic region *	US	96,106 (86.5%)	55,702 (55.7%)	<0.001
	Europe	6234 (5.6%)	16,772 (16.8%)	
	Asia	2250 (2.0%)	6722 (6.7%)	
	Others	6515 (5.9%)	20,894 (20.9%)	
Reporter type	Consumer	94,373 (84.9%)	49,277 (49.2%)	<0.001
	Physician	7191 (6.5%)	21,964 (21.9%)	
	Pharmacist	2892 (2.6%)	8016 (8.0%)	
	Others	6649 (6.0%)	20,833 (20.8%)	
Indication: Obesity	Yes	3609 (3.2%)	336 (0.3%)	<0.001
Indication: Diabetes	Yes	37,132 (33.4%)	56,327 (56.3%)	<0.001
Treatment duration (days)	Mean ± SD	318.38 ± 604.72	972.32 ± 1883.31	<0.001
No. of concomitant drugs	Mean ± SD	0.79 ± 2.68	1.76 ± 4.50	<0.001
Psychotropic co-medication	Yes	3823 (3.4%)	4069 (4.1%)	<0.001
Serious adverse event	Yes	15,402 (13.9%)	39,516 (39.5%)	<0.001
	Death	1224 (1.1%)	7672 (7.7%)	<0.001
	Hospitalization	13,859 (12.5%)	32,890 (32.9%)	<0.001
	Life-threatening	1887 (1.7%)	7683 (7.7%)	<0.001

* Geographic region was derived from the reporting country. Europe includes the United Kingdom, France, Germany, the European Union, and Italy; Asia includes Japan and China; Others include Canada, Australia, and all remaining countries. Abbreviations: FAERS, Food and Drug Administration Adverse Event Reporting System; GLP-1, glucagon-like peptide-1; SD, standard deviation.

**Table 2:** Psychiatric AE signals detected for GLP-1 receptor agonists.

Preferred Term (PT)	a *	PRR (95% CI)	ROR (95% CI)	IC (95% CI)	χ^2^
Suicidal ideation	986	2.95 (2.62–3.32)	2.95 (2.62–3.32)	0.62 (0.58–0.67)	353.2
Emotional distress	417	2.37 (2.00–2.80)	2.37 (2.00–2.80)	0.53 (0.45–0.61)	106.8
Mood swings	200	2.41 (1.89–3.08)	2.41 (1.89–3.08)	0.54 (0.43–0.65)	53.1
Bipolar disorder	160	6.88 (4.56–10.38)	6.88 (4.56–10.38)	0.87 (0.79–0.96)	114.37
Depression suicidal	80	2.32 (1.58–3.39)	2.32 (1.58–3.39)	0.52 (0.34–0.70)	19.81
Major depression	74	5.46 (3.16–9.44)	5.46 (3.16–9.44)	0.82 (0.69–0.96)	46.77
Self-injurious ideation	57	2.47 (1.56–3.91)	2.47 (1.56–3.91)	0.55 (0.34–0.76)	15.81
Intrusive thoughts	41	31.44 (6.17–160.31)	31.44 (6.17–160.31)	1.04 (0.96–1.13)	42.67
Anhedonia	39	4.73 (2.33–9.59)	4.73 (2.33–9.59)	0.78 (0.59–0.98)	22.49
Agoraphobia	27	8.93 (2.94–27.16)	8.93 (2.94–27.16)	0.92 (0.74–1.10)	21.86
Emotional poverty	25	57.96 (3.53–952.02)	57.96 (3.53–952.05)	1.07 (0.99–1.14)	27.45
Suicidal behavior	20	4.24 (1.65–10.86)	4.24 (1.65–10.86)	0.75 (0.47–1.04)	10.72
Self esteem decreased	16	7.50 (1.98–28.36)	7.50 (1.98–28.37)	0.89 (0.64–1.14)	12.23
Alcoholism	16	3.41 (1.30–8.95)	3.41 (1.30–8.95)	0.68 (0.33–1.03)	7.02
Anorexia nervosa	14	2.54 (1.01–6.39)	2.54 (1.01–6.39)	0.56 (0.15–0.97)	4.17
Feelings of worthlessness	8	6.44 (1.14–36.53)	6.44 (1.14–36.54)	0.86 (0.49–1.24)	5.86

* Indicates the number of reports for the specific AE (PT) with the drug of interest. Abbreviations: AE, adverse event; GLP-1, glucagon-like peptide-1; PT, Preferred Term; PRR, Proportional Reporting Ratio; ROR, Reporting Odds Ratio; IC, Information Component; CI, Confidence Interval; χ^2^, Chi-square statistic.

**Table 3:** Top-k enrichment performance for detecting psychiatric AE reports.

Top-k	*n*	Recall	PPV	LR+
Top 1%	527	14.8%	9.3%	14.9
Top 5%	2639	38.8%	4.9%	7.8
Top 10%	5279	53.6%	3.4%	5.4

Abbreviations: AE, adverse event; PPV, positive predictive value; LR+, positive likelihood ratio.

## Data Availability

The original data presented in the study are openly available in the FDA Adverse Event Reporting System (FAERS) at https://fis.fda.gov/extensions/FPD-QDE-FAERS/FPD-QDE-FAERS.html (accessed on 17 December 2025).

## Supplementary Materials

The following supporting information can be downloaded at: https://www.mdpi.com/article/10.3390/ph19060953/s1, Table S1, Sensitivity analysis: Disproportionality results after excluding phentermine-containing products from the comparator group; Table S2, Sensitivity analysis: Disproportionality results restricted to healthcare professional reports; Table S3, Joinpoint regression analysis of quarterly psychiatric AE reporting rates; Table S4, Drug terms used to define exposure, comparator, and concomitant groups in FAERS; Table S5, The 2 × 2 contingency table and formulas for disproportionality analysis using the active comparator cohort; Table S6, Definitions of features used in the machine learning models (n = 23); Table S7, Hyperparameters and specifications of machine learning models used in the study; Table S8, Categorization of psychiatric Preferred Terms (PTs); Figure S1, Time-series trend of quarterly psychiatric AE reporting rates (per 1,000 cases) for GLP-1 receptor agonists and active comparators; Figure S2, Receiver operating characteristic (ROC) curves for machine learning models in the sensitivity analysis (excluding psychotropic co-medication); Figure S3, SHAP summary beeswarm plot illustrating feature contributions in the XGBoost model for the sensitivity analysis (excluding psychotropic co-medication); Figure S4, Calibration curves for the XGBoost model. (a) Before calibration. (b) After post-hoc calibration using isotonic regression.

## Abbreviations

AE	Adverse Event
AUROC	Area Under the Receiver Operating Characteristic Curve
BIC	Bayesian Information Criterion
CI	Confidence Interval
EMA	European Medicines Agency
FAERS	FDA Adverse Event Reporting System
FDA	Food and Drug Administration
GLP-1	Glucagon-like Peptide-1
IC	Information Component
LightGBM	Light Gradient Boosting Machine
MedDRA	Medical Dictionary for Regulatory Activities
PPV	Positive Predictive Value
PRR	Proportional Reporting Ratio
PT	Preferred Term
QPC	Quarterly Percent Change
ROR	Reporting Odds Ratio
SGLT2	Sodium-glucose cotransporter 2
SHAP	SHapley Additive exPlanations
XGBoost	Extreme Gradient Boosting
