# Supplementary material for: Psychiatric Safety Signals of GLP-1 Receptor Agonists: A FAERS-Based Pharmacovigilance Study with Explainable Machine Learning
Source: Pharmaceuticals (Basel). 2026 Jun 18;19(6):953. doi: 10.3390/ph19060953 (PMC13304840; doi:10.3390/ph19060953)
Supplement: Supplementary file 1 [file pharmaceuticals-19-00953-s001.zip › pharmaceuticals-4219656-supplementary.pdf]

Supplementary Materials

Table S1. Sensitivity analysis: Disproportionality results after excluding phentermine-containing products from the comparator group.

Preferred Term (PT)	a*	PRR (95% CI)	ROR (95% CI)	IC (95% CI)	χ2
Suicidal ideation †	986	3.12 (2.77–3.52)	3.12 (2.77–3.52)	0.65 (0.54–0.75)	379.02
Emotional distress †	417	2.38 (2.01–2.82)	2.38 (2.01–2.82)	0.53 (0.37–0.69)	107.26
Mood swings †	200	2.67 (2.07–3.44)	2.67 (2.07–3.44)	0.58 (0.35–0.82)	61.55
Bipolar disorder †	160	6.89 (4.57–10.39)	6.89 (4.57–10.39)	0.88 (0.61–1.14)	112.95
Depression suicidal †	80	2.32 (1.58–3.39)	2.32 (1.58–3.39)	0.52 (0.15–0.89)	19.06
Major depression †	74	5.47 (3.16–9.45)	5.47 (3.16–9.45)	0.82 (0.44–1.21)	45.40
Self-injurious ideation †	57	2.47 (1.56–3.91)	2.47 (1.56–3.91)	0.55 (0.11–0.98)	14.98
Intrusive thoughts †	41	94.41 (5.81–1534.81)	94.41 (5.81–1534.87)	1.08 (0.57–1.59)	43.60
Anhedonia †	39	4.73 (2.33–9.60)	4.73 (2.33–9.60)	0.78 (0.26–1.31)	21.18
Agoraphobia †	27	8.94 (2.94–27.18)	8.94 (2.94–27.18)	0.92 (0.29–1.55)	20.24
Emotional poverty †	25	58.01 (3.53–952.93)	58.01 (3.53–952.96)	1.07 (0.41–1.72)	25.45
Suicidal behaviour †	20	4.24 (1.65–10.87)	4.24 (1.65–10.87)	0.75 (0.02–1.48)	9.49
Self esteem decreased †	16	7.51 (1.99–28.39)	7.51 (1.99–28.39)	0.89 (0.08–1.70)	10.69
Alcoholism	16	3.41 (1.30–8.96)	3.41 (1.30–8.96)	0.68 (-0.13–1.49)	5.95
Anorexia nervosa	14	2.54 (1.01–6.40)	2.54 (1.01–6.40)	0.56 (-0.31–1.43)	3.33
Feelings of worthlessness	8	6.45 (1.14–36.57)	6.45 (1.14–36.57)	0.86 (-0.27–1.99)	4.43

*number of reported cases.

† Signals detected based on the following criteria: (1) number of reports (a*) >= 3, (2) lower bound of the 95% CI for ROR > 1, and (3) lower bound of the 95% CI for IC (IC025) > 0.

Abbreviations: CI, confidence interval; IC, information component; PRR, proportional reporting ratio; PT, preferred term; ROR, reporting odds ratio.

Table S2. Sensitivity analysis: Disproportionality results restricted to healthcare professional reports.

Preferred Term (PT)	a*	PRR (95% CI)	ROR (95% CI)	IC (95% CI)	χ2
Suicidal ideation †	347	11.29 (9.49–13.44)	11.32 (9.51–13.48)	2.25 (2.07–2.42)	1181.41
Emotional distress †	135	13.34 (9.94–17.92)	13.36 (9.94–17.94)	2.33 (2.04–2.61)	499.64
Depression suicidal †	31	23.90 (11.21–50.99)	23.91 (11.21–51.00)	2.55 (1.96–3.14)	141.33
Mood swings †	15	3.17 (1.73–5.83)	3.17 (1.73–5.83)	1.30 (0.46–2.14)	13.83
Intrusive thoughts †	11	49.45 (9.02–271.09)	49.45 (9.02–271.12)	2.72 (1.75–3.69)	56.70
Bipolar disorder †	11	9.89 (3.94–24.81)	9.89 (3.94–24.82)	2.17 (1.20–3.15)	32.34
Major depression †	10	10.42 (3.92–27.71)	10.42 (3.92–27.71)	2.20 (1.18–3.22)	30.15
Self-injurious ideation †	10	3.66 (1.72–7.81)	3.66 (1.72–7.81)	1.43 (0.41–2.45)	11.07
Anhedonia †	9	11.14 (3.90–31.84)	11.14 (3.90–31.84)	2.24 (1.17–3.31)	28.00
Suicidal behaviour †	8	21.93 (5.35–89.84)	21.93 (5.35–89.85)	2.53 (1.39–3.66)	33.29
Anorexia nervosa †	7	32.25 (5.59–186.15)	32.25 (5.59–186.17)	2.63 (1.43–3.84)	32.07
Emotional poverty †	3	45.15 (2.33–874.12)	45.15 (2.33–874.15)	2.70 (0.94–4.47)	13.05
Self esteem decreased †	3	45.15 (2.33–874.12)	45.15 (2.33–874.15)	2.70 (0.94–4.47)	13.05
Agoraphobia	1	19.35 (0.79–475.01)	19.35 (0.79–475.02)	2.48 (-0.21–5.18)	2.30
Alcoholism	1	6.45 (0.67–62.01)	6.45 (0.67–62.01)	1.90 (-0.80–4.59)	1.02

*number of reported cases.

† Signals detected based on the following criteria: (1) number of reports (a*) >= 3, (2) lower bound of the 95% CI for ROR > 1, and (3) lower bound of the 95% CI for IC (IC025) > 0.

Abbreviations: CI, confidence interval; IC, information component; PRR, proportional reporting ratio; PT, preferred term; ROR, reporting odds ratio.

Table S3. Joinpoint regression analysis of quarterly psychiatric AE reporting rates.

Group	Joinpoints (n)	BIC	Segment	QPC (%)	95% CI	p-value
GLP-1 receptor agonists	1	54.96	2021 Q2–2023 Q4	+19.56	9.45, 29.66	0.002
			2023 Q4–2025 Q3	−2.80	−19.17, 13.56	0.742
Comparator group	0	2.50	2021 Q2–2025 Q3	−0.42	−2.58, 1.74	0.707

Abbreviations: QPC, quarterly percent change; CI, confidence interval; BIC, Bayesian Information Criterion.

Table S4. Drug terms used to define exposure, comparator, and concomitant groups in FAERS

Group	Drug class	Included generic and product name terms (examples)
Exposure (GLP-1 RA)	GLP-1 receptor agonists / dual agonist	Semaglutide (OZEMPIC, WEGOVY, RYBELSUS); Liraglutide (VICTOZA, SAXENDA); Dulaglutide (TRULICITY); Exenatide (BYETTA, BYDUREON); Lixisenatide (ADLYXIN, LYXUMIA); Tirzepatide (MOUNJARO, ZEPBOUND)
Comparator	Biguanide	Metformin
	Insulin and insulin analogues	Insulin (generic term) and common analogs/products: insulin glargine (LANTUS/TOUJEO/BASAGLAR), insulin degludec (TRESIBA), insulin detemir (LEVEMIR), insulin lispro (HUMALOG), insulin aspart (NOVOLOG/NOVORAPID/FIASP), insulin glulisine (APIDRA)
	SGLT-2 inhibitors	Canagliflozin; Dapagliflozin; Empagliflozin; Ertugliflozin
	DPP-4 inhibitors	Sitagliptin; Saxagliptin; Linagliptin; Alogliptin; Vildagliptin
	Sulfonylureas	Glimepiride; Glipizide; Glibenclamide/Glyburide; Gliclazide
	Anti-obesity agents	Orlistat (XENICAL, ALLI); Phentermine-containing products (PHENTERMINE, QSYMIA; phentermine/topiramate combinations)
Concomitant medications	Psychotropic agents	Antidepressants: Fluoxetine, Sertraline, Paroxetine, Citalopram, Escitalopram, Venlafaxine, Duloxetine, Desvenlafaxine, Bupropion, Mirtazapine
		Anxiolytics: Alprazolam, Lorazepam, Diazepam, Clonazepam, Buspirone
		Antipsychotics: Risperidone, Olanzapine, Quetiapine, Aripiprazole, Haloperidol
		Mood stabilizers: Lithium, Valproic Acid, Lamotrigine, Carbamazepine

Abbreviations: FAERS, FDA Adverse Event Reporting System; GLP-1 RA, glucagon-like peptide-1 receptor agonist; GIP, glucose-dependent insulinotropic polypeptide; SGLT-2, sodium–glucose cotransporter-2; DPP-4, dipeptidyl peptidase-4.

Table S5. The 2×2 contingency table and formulas for disproportionality analysis using the active comparator cohort.

	Event of Interest (Target PT)	All other Events	Total
GLP-1 RAs	a[1]	b[2]	a+b
Active Comparator Agents	c[3]	d[4]	c+d
Total	a+c	b+d	N

1) Number of cases in which GLP-1 RAs and the adverse event of interest were reported. 2) Number of cases in which GLP-1 RAs were reported but the adverse event of interest was absent. 3) Number of cases in which Active Comparator Agents and the adverse event of interest were reported. 4) Number of cases in which Active Comparator Agents were reported but the adverse event of interest was absent. Abbreviations: GLP-1 RAs, glucagon-like peptide-1 receptor agonists; PT, preferred term.

Table S6. Definitions of features used in the machine learning models (n = 23).

Variable Name	Definition
Demographics & Reporter	
sex_F	Female sex (1 = yes, 0 = otherwise)
sex_M	Male sex (1 = yes, 0 = otherwise)
age_grp_19–44	Age group 19–44 years (1 = yes, 0 = otherwise)
age_grp_45–64	Age group 45–64 years (1 = yes, 0 = otherwise)
age_grp_65+	Age group ≥65 years (1 = yes, 0 = otherwise)
age_grp_≤18	Age group ≤18 years (1 = yes, 0 = otherwise)
reporter_type_CONSUMER	Report submitted by consumer (1 = yes, 0 = otherwise)
reporter_type_PHARMACIST	Report submitted by pharmacist (1 = yes, 0 = otherwise)
reporter_type_PHYSICIAN	Report submitted by physician (1 = yes, 0 = otherwise)
reporter_type_OTHER	Report submitted by other reporters (1 = yes, 0 = otherwise)
Clinical Characteristics	
CONCOM_GRP_0	No concomitant medications (1 = yes, 0 = otherwise)
CONCOM_GRP_1–2	1–2 concomitant medications (1 = yes, 0 = otherwise)
CONCOM_GRP_3–4	3–4 concomitant medications (1 = yes, 0 = otherwise)
CONCOM_GRP_≥5	≥5 concomitant medications (polypharmacy) (1 = yes, 0 = otherwise)
NUM_PSYCH_CONCOM	Number of concomitant psychotropic medications (count)
CONCOMITANT_PSYCH_DRUG	Any concomitant psychotropic drug use (1 = yes, 0 = no)
INDI_OBESITY	Obesity reported as indication (1 = yes, 0 = no)
INDI_DIABETES	Diabetes reported as indication (1 = yes, 0 = no)
Exposure Variables	
IS_SEMA	Semaglutide (Ozempic, Wegovy, Rybelsus) reported as suspect drug (1 = yes, 0 = no)
IS_LIRA	Liraglutide (Victoza, Saxenda) reported as suspect drug (1 = yes, 0 = no)
IS_TIRZ	Tirzepatide (Mounjaro, Zepbound) reported as suspect drug (1 = yes, 0 = no)
IS_DULA	Dulaglutide (Trulicity) reported as suspect drug (1 = yes, 0 = no)

IS_OTHER	Exenatide (Byetta, Bydureon) or lixisenatide (Lyxumia, Adlyxin) reported as suspect drug (1 = yes, 0 = no)

Table S7. Hyperparameters and specifications of machine learning models used in the study.

Model	Hyperparameter	Value	Description
Logistic Regression	penalty	l2	Regularization norm (standard).
	C	0.001	Inverse of regularization strength.
	solver	lbfgs	Optimization algorithm.
	class_weight	balanced	Automatically adjusts weights inversely proportional to class frequencies.
	max_iter	5000	Maximum number of iterations for convergence.
	random_state	92	Seed for reproducibility.
XGBoost	n_estimators	200	Number of gradient boosted trees.
	learning_rate	0.03	Boosting learning rate.
	max_depth	4	Maximum tree depth for base learners.
	min_child_weight	7	Minimum sum of instance weight (hessian) needed in a child.
	subsample	0.8	Subsample ratio of the training instances.
	colsample_bytree	0.8	Subsample ratio of columns when constructing each tree.
	scale_pos_weight	159	Control the balance of positive and negative weights.
	tree_method	hist	Histogram-based algorithm for efficiency.
	random_state	92	Seed for reproducibility.
LightGBM	n_estimators	300	Number of boosted trees to fit.
	learning_rate	0.03	Boosting learning rate.
	max_depth	4	Maximum tree depth for base learners.
	min_child_weight	7	Minimum sum of instance weight needed in a child (leaf).
	subsample	0.8	Subsample ratio of the training instances.
	colsample_bytree	0.8	Subsample ratio of columns when constructing each tree.
	scale_pos_weight	159	Weight of positive class in binary classification.
	random_state	92	Seed for reproducibility.

Note: Hyperparameters for C, n_estimators, learning_rate, and max_depth were optimized via 5-fold

cross-validated grid search on the training set. Identical settings were applied to the sensitivity analysis.

Abbreviations: XGBoost, Extreme Gradient Boosting; LightGBM, Light Gradient Boosting Machine.

Table S8. Categorization of psychiatric Preferred Terms (PTs)

Category (SOC)	Preferred Term (PT)
Psychiatric disorders	Suicidal ideation, Suicidal behaviour, Depression suicidal, Self-injurious ideation, Major depression, Anhedonia, Feelings of worthlessness, Emotional poverty, Self esteem decreased, Emotional distress, Mood swings, Bipolar disorder, Agoraphobia, Intrusive thoughts, Alcoholism, Anorexia nervosa

Note: Medical Dictionary for Regulatory Activities (MedDRA) terminology was applied for the classification of preferred terms (version 27.0).
Abbreviations: SOC, System Organ Class; PT, Preferred Term.

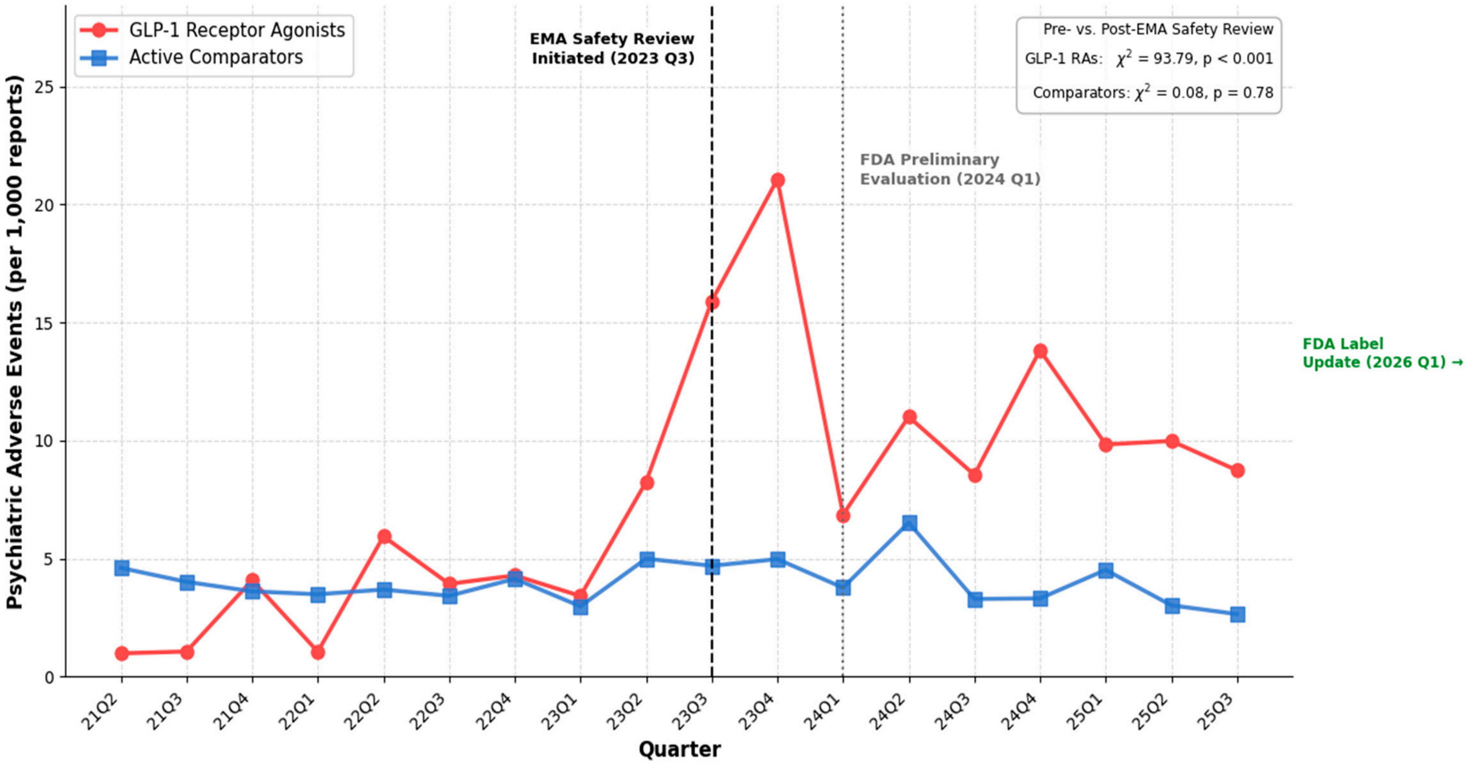

Figure S1. Time-series trend of quarterly psychiatric AE reporting rates (per 1,000 cases) for GLP-1 receptor agonists and active comparators.

Note: Dashed vertical line indicates the initiation of the EMA safety review in 2023 Q3. Dotted vertical line indicates the FDA preliminary evaluation in 2024 Q1. Chi-square comparison of pre-EMA and post-EMA periods: GLP-1 RAs $\chi^2 = 93.79$, $p < 0.001$; comparators $\chi^2 = 0.08$, $p = 0.78$.

Abbreviations: GLP-1 RA, glucagon-like peptide-1 receptor agonist; EMA, European Medicines Agency; FDA, Food and Drug Administration; Q, quarter.

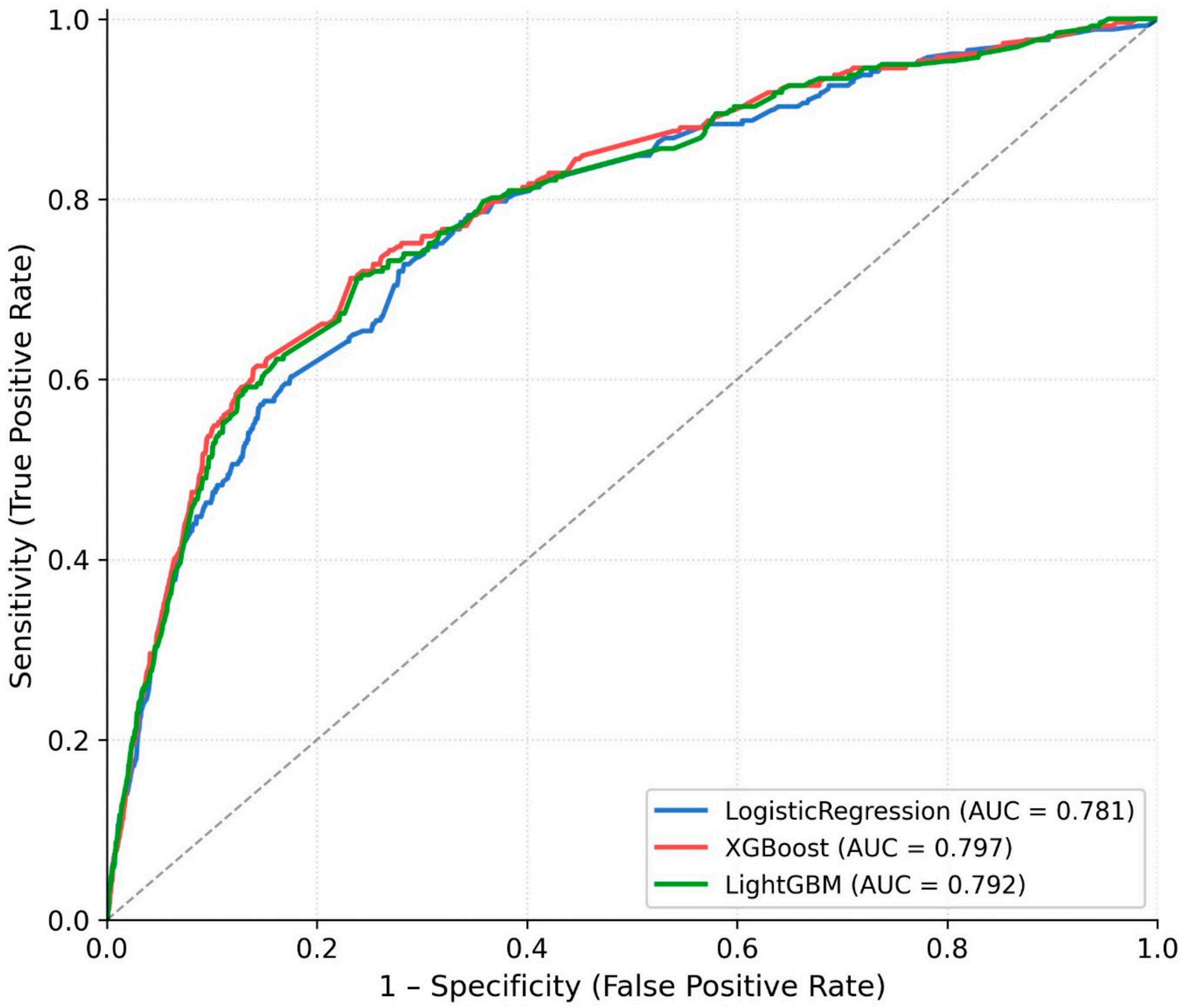

Figure S2. Receiver operating characteristic (ROC) curves for machine learning models in the sensitivity analysis (excluding psychotropic co-medication).

Abbreviations: ROC, receiver operating characteristic; AUROC, area under the receiver operating characteristic curve; XGBoost, extreme gradient boosting; LightGBM, light gradient boosting machine.

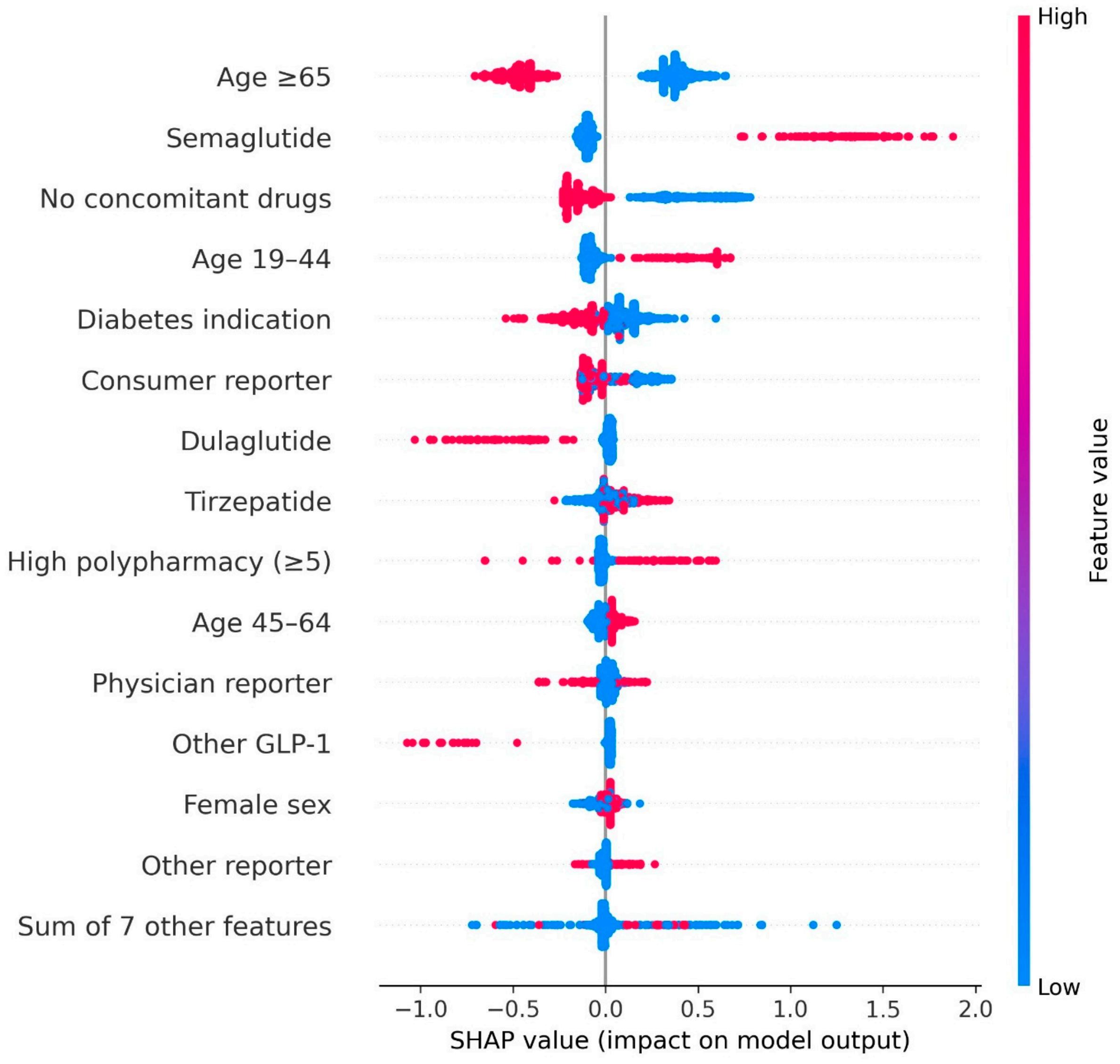

Figure S3. SHAP summary beeswarm plot illustrating feature contributions in the XGBoost model for the sensitivity analysis (excluding psychotropic co-medication).

Abbreviations: SHAP, Shapley Additive Explanations; XGBoost, extreme gradient boosting.

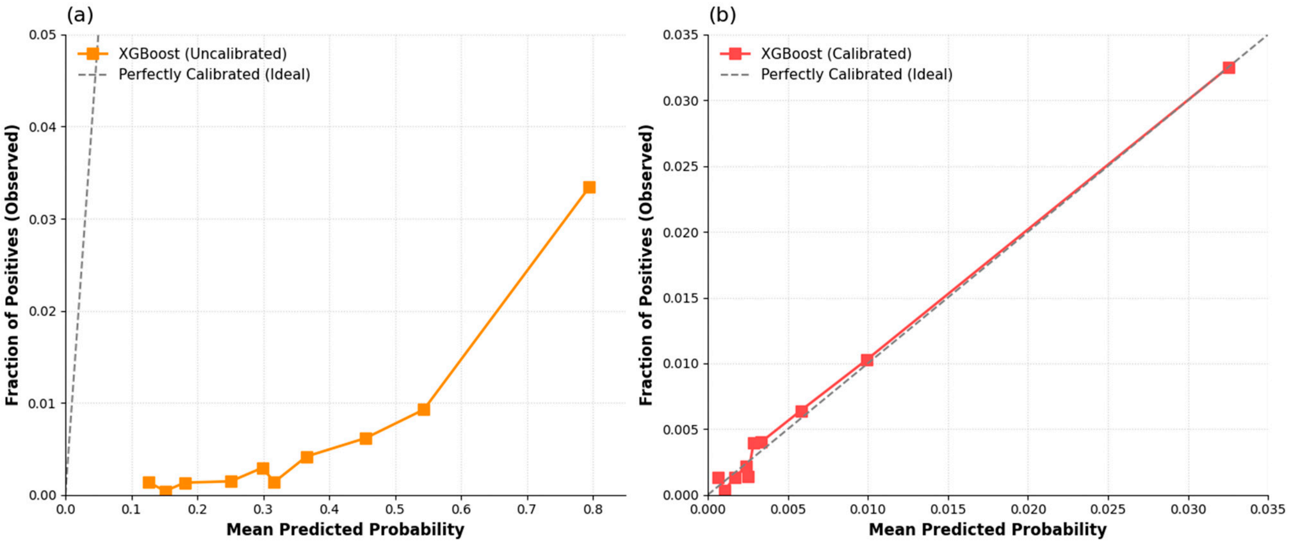

Figure S4. Calibration curves for the XGBoost model.
(a) Before calibration. (b) After post-hoc calibration using isotonic regression.
Abbreviations: XGBoost, extreme gradient boosting.

Note: axis scales differ between panels to reflect the range of predicted probabilities before and after calibration.
